# Analectic

**Published:** 1828-10

**Authors:** 


### MISCELLANEOUS INTELLIGENCE.

---

### I.—ANALECTIC.

1. *Ligature in Nœvi Materni.*—Mr. Lawrence, surgeon to St. Bartholomew's hospital, communicates a number of cases of this nature successfully treated by ligature by himself and Mr. White, surgeons to the Westminster hospital, from whom he learned this mode of treatment. The cases to which it is applicable, together with the mode of operating, may be learned from the following case, selected from a considerable number.

*Case. I.*—J. B. an infant four months old, was brought to St. Bartholomew's Hospital in February, 1827.

At the time of birth, a slight reddish discoloration of the skin, as large as a sixpence, was observed at the upper part of the back; this red spot was not elevated. Having remained of the same size and in the same state for three months, it began to enlarge, became elevated above the surrounding sound skin, changed its colour from red to a dull purple, and frequently discharged blood from openings on its surface. It continued to grow rapidly until the time of admission, when it presented a circular tumour five inches and a half in circumference, and projecting about three quarters of an inch. Its surface was slightly irregular, of nearly uniform prominence, and a little depressed in the middle; it was rather larger at the edge than at the base, which rendered the application of the ligature easy. It was of the kind denominated by Mr. Wardrop, subcutaneous nævus. Although the skin had been distended and thus rendered thin by the growth of the tumour, it could be easily pinched up into folds on the surface, so that the disease was decidedly below the integuments. Its connexion to the muscles of the back was equally loose, and it could be moved from side to side with the greatest ease, or drawn up by the finger and thumb, and detached from the subjacent parts. It had a mottled appearance, being mostly of a dark livid colour.—It was soft, and gave a sensation to the fingers like a collection of varicious vessels. Pressure diminished it considerably, and made it of lighter colour; it soon filled again. When the child cried or strained, it became larger and of a deep purple tint.

On the 17th of February I tied the tumour, elevating it from the muscles of the back with one hand, while I passed a large curved needle under the middle of its basis with the other, carrying the point of the needle in and out again, close to the edge of the base of the swelling. When the needle had been cut out, the two ends of the ligature, which had been left of equal length, formed two distinct ligatures applicable to the immediate purpose of the operation. They were then tied, as tightly as they could be drawn, just in the line of distinction between the tumour and the sound skin. The firm pressure thus made on the base caused a cracking of the tumour at the surface, from which a few drops of arterial blood escaped before the ligature was quite tight. The infant seemed to suffer considerably when the ligatures were tied, the process being attended with considerable dragging and stretching of the surrounding skin, which was thrown into several large folds. A soft rag dipped in cold water was laid over the part, and occasionally moistened. For thirty-six hours the child was restless, crying almost incessantly and occasionally convulsed; at the end of that time it took the breast, which it had previously refused. An inconsiderable oozing of blood took place. On the 9th I sliced off the tumour, which had become almost black. The ligatures were quite loose on the 20th, and I removed them. A large slough occupied the centre of the wound, extending deeply; it came away in a few days under the application of bread poultice. The wound then granulated and assumed a perfectly healthy appearance, all the unpleasant symptoms having subsided after the removal of the ligatures. The process of cicatrization was slow, but it was at last firmly completed; and the former situation of this large tumour is now denoted by a very slight scar.

The substance of the tumour, when it was sliced off on the 19th, was tolerably compact: its cut surface presented the sections of numerous blood-vessels, apparently venous, filled with coagulated blood: the largest were equal to an ordinary writing quill. These vessels were connected by a firm grayish substance, of a somewhat fibrous texture. There was no appearance of cells.

What is the nature of such vessels? The colour of the part during life, the size of the tubes, and the appearance of their coats lead us to suppose that they are veins. How then does it happen that the profuse bleeding, which takes place when the tumour is cut, is arterial? And how is it that the growth of such structures is arrested, and their absorption produced by tying the principal artery of the part?—*Amer. Med. Rec. No.* 44.

2. *Acupuncture in Rheumatism* has been extensively practised by Dr. Elliott, in private and at St. Thomas's Hospital. He thinks one needle allowed to remain an hour or two in a part, is more efficient than several used but for a few minutes.

Three or four applications to each painful part are generally required, and he has known the disease not yield until the ninth.

Out of forty-two cases taken from the hospital books in succession, thirty were cured.—*Ibid.*

3. *On the Treatment of Colica Pictonum.* [*By M. M. Chevalier and Rayer.*]—Three principal indications present themselves in the treatment of poisoning caused by the salts and oxides of lead, and particularly in the colic produced by that metal. The first is to neutralize the poison by the internal administration of hydrosulphurous water, in quantities proportioned to the known or presumed quantity of the noxious matter which has been received into the system. M. Rayer has employed the water of Enghien in his experiments with success. An artificial water, prepared according to the formulæ, No. 1 and 2, may also be employed.

No. 1. Take eight gallons of water, and thirty-five fluid ounces of water saturated with hydrosulphuric acid, to which has been added ten grains of the subcarbonate of soda previously to the saturation. Mix.

No. 2. Take four grains of sulphuret of potass, and dissolve it in thirty-five fluid ounces of water.

The good effects of these fluids are in an inverse ratio to the time which has elapsed since receiving the poison. Many obstinate cases of colic from lead have rapidly recovered under this treatment.

The second indication is to combat constipation when it exists. This is accomplished by purgatives of a strength proportioned to the degree of the constipation. Pills of equal quantities of jalap and scammony should be repeated until copious evacuations be produced. In cases of obstinacy, a lavement of infusion of senna and caster oil should be administered.

The third indication is to allay the pain and to procure sleep: for which purpose eight or ten drops of laudanum of Rousseau, or a grain and a half of opium, should be given.

Under this plan of treatment, the disease rapidly disappears, sometimes on the second day, often on the third or fourth, and it rarely continues beyond the sixth.

M. Rayer has never seen a relapse, although he has thought proper to retain some of the patients in the hospital for several days after their recovery.—*Journal de Clinique Medicale.—Ibid.*

4. *Obstruction on the arteries.*—This was a case of gangrene of the left leg, arising without any obvious cause—the circumstances of the case led to the operation of amputation, which was performed by Mr. Syme. The patient died in a short time after, and on dissection the right internal iliac artery was found entirely obstructed from its origin, as were also the right and left internal iliac veins. The left femoral was pervious to the ligature, but the profunda and corresponding vein were firmly obstructed, also the same vessels on the right side. The whole of the arteries appeared to be in a healthy condition as to texture. Mr. Syme's opinion of the case is, that it was one of acute inflammation of the arteries, and in conclusion, ventures to suggest "that obstruction of arteries, as a consequence of effusion from their own coats, may not be so rare an occurrence as it would seem to be, from the extreme rarity of such cases on record."

5. *Encephalitis cured by Acupuncturation.*—G. Grassi, after being for a long time subject to frequent fits of dulness, caused by cerebral congestions, and which was removed by bleeding and purging, was seized with apoplexy, with hemiplegia, loss of voice, grinding of the teeth, irregular breathing, intermittent and sometimes scarcely perceptible pulse, without any signs of gastro-intestinal irritation. Repeated bleedings and purges brought him back to a pretty good state of health. At the end of some months all symptoms of local affection had disappeared; the pulse was regular; the voice a little returned, as well as the movements of the affected limbs in part, when Dr. Strambio proposed electro-puncturation to complete the cure. Dr. Fantonelli performed this operation, which was done by inserting a needle at the lower part of the neck, on the side opposite to that affected; then another needle into the external ankle of the affected limb; a metallic wire, communicating with two needles, was connected with a voltaic pile of five plates only. The introducing of the needles was not at all painful, but at each shock severe pains and violent contractions were induced in the muscles nearest to the needles, and chiefly in those of the affected side, (at the negative pile the pain was greatest.) After five or six shocks it was stopped, as the pain was intolerable.

The electro-puncturation was repeated three times, with intervals of one day. After the first, the patient felt himself livelier, and moved more freely. At the second trial, he felt some inconvenience; and, on the third, he was seized with violent fever and symptoms of cerebral congestion. Bleedings and revulsives soon calmed this encephalitis, but his former ailments became just as they were before the trials were attempted.—*Giornale Critica di Med. Analitica, Milano.—Ibid.*

6. *Wounds and Ulcers cured by plates of Lead.*—In all wounds, the simpler the treatment the better. This principle of surgery is now so generally recognised, that there remain few practitioners who have not laid aside all multiform and compound applications to sores, and which only retard their cure. Quiet, cleanliness, dressings with lint, either dry or spread with salve, form the basis of treatment now followed, unless in very particular cases. M. Reveille Parise has suggested a still simpler mode of treatment. In a Memoir read some time since at the Academie de Medicine, he states that he had derived the most advantageous results from plates of lead applied to wounds. Guy de Chauliac and Ambrose Pare had before employed, in the cure of ulcers, plates of lead rubbed with mercury; but this means, neglected by men of science, passed into the hands of the vulgar, where it is still occasionally met with. The following fact will show this.

A military invalid had applied to his ulcers a plate of lead: surprised to observe that they regarded his being dressed by this means as a new fact, he said that, twenty years before, having received a wound on the field of battle, he cured himself by applying a leaden bullet that he beat out with a flint. He said that this remedy was known to many soldiers, who always tried it when their wounds were not sufficiently bad to go into hospital.

M. Parise, however, has not the less merit in restoring to science a mode of cure which may be very useful. Recent wounds are almost the only ones in which he has tried his plan. His success induced M. le Baron Yvans to repeat the trials; and the great number of people affected with ulcers in the establishment at the head of which he is, gave him great facility to make the experiment.

M. Menon has not seen a sufficient number of recent wounds tried with this means, to speak of its effect; but more than sixty cases of ulcers successfully treated in this way convince him that the plates of lead form the best topical application to ulcers. Among these cases, some have been of thirty years' standing. When they were not completely cured, the ulcer quickly put on a new aspect; its edges thickened, diminished in size, and soon assumed a more satisfactory appearance. It was remarked that several ulcers, which had never been able to be cicatrised, were so by this new method, and that those in which it failed were never cured by any other means: and we may conclude that these last were incurable, and that nature, more powerful than art, resisted the suppression of an evacuation that custom had rendered necessary. At first this method was confined to simple ulcers: emboldened by success, M. Yvans tried it on ulcers evidently in an inflammatory state, and which before he would have treated by topical emollients. He was astonished to see that the lead did not only not increase pain, but the inflammatory symptoms diminished, and shortly the ulcer was in a healthy state. In fact, many cases prove that this substance acts with a promptitude quite surprising in bringing ulcers, foul at bottom and having all those appearances that are implied by the name "ulceres sordides," back to a healthy state. Three or four days have in general been sufficient, and soon after cicatrization has generally begun to take place.

The fact being established that lead, in the form of plates, applied to wounds is beneficial, it would be interesting to know how it acts. Does this lead enjoy any specific property? Can it have within it something peculiar which, changing the kind of irritation of the ulcerated surface, disposes it to heal? Is there any chemical action takes place, from which might result a compound analogous to the acetate of lead which Goulard and others have so much praised? M. Menon thinks not; for it appears that plates of brass, or gold, or silver, have nearly the same results. Lead is, however, preferable, both being cheaper and also more flexible, so that it is modelled more exactly to the surface. M. Menon thinks it acts mechanically, exercising slight compression on the borders of the wound, and keeping in contact with it the pus which many physiologists think necessary to the formation of a cicatrix.

This mode of dressing consists in the simple application of a thin plate of lead on the ulcer, and keeping it in its place by a bandage. When it is wished to be removed, the surface of the ulcer and the plate are both wiped clean, and again brought into contact. Nothing can be simpler; and it is a great advantage, not exposing the surface of the sore too long to the action of the air. If granulations shoot up too fast, touch them with lunar caustic, or powder them with calcined alum.—*London Med. and Phys. Journal.*

7. *Remarkable disease of the Epiploon.*—The following melancholy case is recorded by Dr. Strambio, in an Italian Journal, (*Annali di Med.*) with the appearances on dissection.

A young woman 18 years of age, after enjoying herself at a carnival ball, began to complain of pains in the right side of the abdomen, which increased in size, and led to the suspicion that she was pregnant. Various medicines were ineffectually administered, and when M. Strambio was consulted, the patient presented the following phenomena:—face pale—pulse hard and frequent—skin burning hot—vomiting when food was taken into the stomach—abdomen large, but more so in the right than in the left side. On examination, several indolent tumours were felt, which were not painful unless strongly pressed. The left mamma was shrunk, and the right more enlarged than natural, as well as hard. There was supposed to be inflammation of the stomach, and depletion was practised, locally and generally, with fomentations, &c. It was found that a tumour pressed on the rectum, and prevented the introduction of a pipe, or the finger. All the symptoms increased in severity, and hydrothorax supervened, with infiltration of the left lower extremity. Death soon took place.

On dissection, a large effusion was found in the chest and also in the pericardium. The omentum, detached from the viscera to which it naturally adheres, was degenerated into an elastic substance, resembling brain rather than fat, occupying the whole of the abdomen, and disposed into masses of various sizes, connected by prolongations of the original omentum or membranous strips. The left kidney, the spleen, the abdominal aorta, the rectum, the ovaries, and the uterus, were involved in this morbid mass. The other organs were unaltered. The disease was considered to be caused by a chronic phlegmasia of the epiploon, excited by a fall which the young woman had experienced about a year before her death.—*Medico Chir. Rev. for* 1828.

8. *Laryngitis.*—In a short clinical report, published by Dr. Martinet, in a late number of the Revue Medicale, an interesting case is detailed, of which we shall give the particulars in this place.

1. *Angina Suffocans.* The author avoids entering into the discussion, whether croup be a disease essentially different from cynanche laryngea et trachealis. The angina suffocans is an inflammation about the top of the air-passage, and presents some differences, according as the inflammation is seated in one or other part of the larynx or its appendages. The alteration of the voice, the sound of the cough, the distressing paroxysms of suffocation, the kind of expectoration, (when there is expectoration,) suffice to characterize *cynanche laryngea and cyn. trachealis;* while, on the other hand, the tumefaction and redness of the velum palati, tonsils, and pharynx, together with the presence of crusts or exudations of different sizes, on these parts—the difficulty, or even impossibility, of swallowing—the regurgitation of liquids through the nose, these will readily mark the existence of cynanche pharyngea and tonsillaris. Œdema of the glottis may be suspected, when there is a sensation as if a foreign body were placed in the throat, and more especially when by the finger, a kind of ring or ridge is felt surrounding the rima glottidis—attended with extreme dyspnœa. But often there is a complication of all these affections in the same subject.

In some instances, the angina suffocans does not show unequivocal characters of *inflammation*, and then our practice is necessarily embarrassed—some trusting to depletion, some to calomel, and others to emetics and counter-irritation. In this state of vacillation as to the best method of treatment, the patient often dies by the rapidity of the disease, and the derangement which some of the most vital functions in the body experience. The following case will show the path which we ought to pursue, where the subject is young and vigorous. It will also demonstrate the power of art over one disease at least.

*Case.* Peter Chapion, 26 years of age, of vigorous and sanguineous constitution, came into the Hotel Dieu on the 11th March, being that day seized with acute pain in the throat, about the top of the larynx, quickly followed by difficulty of breathing, and sense of suffocation. Eight ounces of blood were taken from the arm, but the sense of suffocation continued, and made progress, without increase of frequency in the pulse, or elevation of heat on the surface. *Thirty leeches* were applied round the neck, and, the same evening, venesection was repeated to *twenty ounces.* The dyspnœa was relieved by this last bleeding, and the sense of suffocation rendered less imminent. *Mustard pediluvia.* 12*th.* The pain at the top of the throat is still acute—the voice is altered from its natural tenor, being much weaker—respiration difficult and sonorous, but not accompanied with much sense of suffocation—tumefaction of the tonsils and uvula—no appearance of albuminous exudation on any part of the fauces—the deglutition is difficult—pain on pressure of the larynx. Some of the physicians, on introducing the finger, thought they felt a swelling about the rima glottidis. There was an expuition of reddish and sanguinolent saliva—pulse easily compressible and very little increased in frequency—heat moderate– countenance indicative of anxiety—great prostration of strength—face neither pale nor flushed, but the circulation apparently impeded—inability to deviate from the upright posture. *Venesection—blister to the nucha—purgatives—sinapisms to the lower extremities.* This day passed, like the preceding, in a state of constant dyspnœa, with occasional paroxysms of sense of suffocation, not, however, so threatening as to require tracheotomy. *Sixty leeches were applied round the neck*, and a cataplasm over the bites. 13*th.* The sense of suffocation is diminished, and the patient can fill the chest, by a deep inspiration—expectoration very difficult, but changing in character to the muco-purulent form. *Twenty more leeches to the neck.* On examination with the stethoscope, the breathing was heard much more distinctly in one side of the chest than in the other, and the *rale muqueux* indicated inflammatory action of the mucous membrane of the bronchia. From this time, the expectoration became freer, and the dyspnœa less, until convalescence was established.

*Remarks.* The above is a good sample of that dangerous disease laryngitis. The depletion, except in the first instance, was bold and decided; but the Hotel Dieu physicians deprived themselves of a powerful means of checking inflammatory action, by withholding nauseating doses of antimony and calomel, which increase the intestinal secretions, and save a great deal sanguineous depletion.—*Ibid.*

9. *Enlargement of the Upper Lip.*—In a recent number of the Journal de Progres, Dr. Paillard has drawn the attention of his brethren to the treatment of this peculiar deformity. He observes that this said enlargement is generally considered as an effect, as well as a sign of scrofula. It usually appears in scrofulous subjects, and in that period of life when scrofula prevails—and it generally disappears when the scrofulous diathesis ceases, or is overcome. But we occasionally observe this phenomenon where there is no scrofula in the constitution, and it sometimes remains as a striking deformity, after the other symptoms of scrofula have entirely vanished.

If, in a scrofulous subject, we examine this swelling of the upper lip, we find the cellular tissue more abundant than natural, and infiltrated with a serous fluid—the muscles more pale and flabby than in a healthy subject—the skin very pallid or shining, and, as it were, infiltrated. Sometimes this swelling is covered with ulcerations, which discharge a matter that forms into scabs, which fall off, and are renewed from time to time. The cellular tissue, then, appears to be the seat of this affection. But the treatment differs, according as the complaint is an indication of scrofula, or independent of that diathesis. In the *former* case, local treatment is of no avail, and we must cure the local complaint through the medium of the constitution. In the *latter* case, the local treatment now to be mentioned is efficacious. This operation consists in an incision from one angle of the mouth to the other, along the inner side of the upper lip, and thus, in fact, dissecting away a mass of condensed cellular tissue forming the swelling. The hæmorrhage is very great; but it either ceases spontaneously, or may be restrained by ligature of the bleeding vessels. The operation is very painful, but not dangerous, and it removes a deformity which gives a peculiarly stupid cast of countenance to the individual afflicted with it. For the cases detailed in illustration of the above proceeding, we must refer to the Journal already quoted, vol. 3, 1827.—*Ibid.*

10. *Baron Larrey on Fractures.*—By a private communication, we learn that the above mentioned veteran has projected and makes use of, an extraordinary dressing for fractured limbs. He encircles the whole of the member with compresses and bandages soaked in gummy or albuminous substances, which, on drying, form a complete immoveable, and inflexible case for the injured limb. This he applies, whether the fracture be simple or compound, and never takes it off till the cure is completed, whatever may be the degree of swelling, infiltration, or even suppuration, that supervenes. The swelling is either prevented, or subsides, without danger—the infiltration or suppurations are absorbed—and the process of re-union goes on safely. We think it not improbable that, in simple fractures this plan may have some advantages over the splints and bandages now in use. Baron Larrey's apparatus forms a complete shell or mould that adapts itself to every part of the limb's surface, and thus forms a more permanent and imperturbable case for the member, than any apparatus which can be constructed of splints. There is this advantage, also, that the member is never disturbed by subsequent examinations or adjustments—a frequent source of displacement of the bones. But how far this apparatus will apply to compound fractures, especially where there is much laceration of the soft parts, or shattering of the bones, we will not venture to say. We should suppose that it never can become generally applicable in such cases. We understand, however, that some of the Parisian surgeons are adopting the plan of the Baron.—*Vide Journal de Progres, vol.* iv.—*Ibid.*

11. *Physiological and Pathological results of extirpation of the Kidneys.*—[Professor Mayer, of Bonn.]—Passing over the crude speculations of the ancients, respecting the functions of the kidneys, and the role which this function occupies in the animal economy—passing over, also, the instances in which these organs were found wanting in fœtuses and in monsters, we shall come at once to modern times, when experiments have been made with care, and their effects accurately noted.

When Richerand extirpated one kidney of an animal, no inconvenience appeared to ensue; but, when both kidneys were removed, a morbid condition obtained, and death took place in a very few days. In all Richerand's experiments, the gall-bladder was found gorged after death. The principal phenomena which succeeded the ablation of both kidneys, were—Vomiting, tremors, smallness of pulse, urinous odour in the liquids vomited, borborigmi, intermissions of the pulse, coldness of the body—death in three days. On dissection, some effusion was found in the abdomen, but no inflammation; venous system gorged with blood—no alteration in the chest—slight effusion into the cerebral ventricles. In some cases, the animal died in a quarter of an hour after the extirpation, and nearly the same symptoms and *post-mortem* appearances presented themselves.

Prevost and Dumas made similar experiments, and they discovered the presence of urea in the blood, after renal ablation. M. Mayer made a number of experiments, of which he has detailed ten, in a late number of a French journal. We shall only give the results, and not the details.

1. The extirpation of both kidneys causes inevitable death of the animal, at various intervals.

2. The principal phenomena observed were tremors; crying apparently from internal pains; and, finally, convulsions and death.

3. There were no well-marked symptoms of abdominal inflammation.

4. The operation is followed by the secretion, in various organs of the body, of a fluid, having all the physical characters of urine.—This secretion takes place, particularly in the abdomen, chest, pericardium, ventricles of the brain, the eye. stomach, and intestinal canal. It even takes place in the cellular tissue of the liver, lungs, muscles, testicles, &c.

This urinous serum was submitted to chemical analysis, and our author's experience coincided with that of Prevost and Dumas, who also detected urea in the blood of animals, after ablation of the kidneys, corresponding with the fact, that men, whose kidneys have been affected with organic disease, have vomited up matters clearly of a urinous character. We cannot, then, says our authors, deny, that a urinous liquid may be formed, under the above circumstances, in various other parts, besides the kidneys—but particularly in secreting structures.

Dr. Mayer thinks it probable that the cause of death, after these experiments, is owing to the irritation of the brain, from the urinous fluid thrown out there.—*Journal Complementaire.—Ibid.*

12. *Section of the Pneumo-Gastric Nerves.* [M. Dupuy. Veterinary School of Alfort.] It is now ascertained, beyond all doubt, that section of the par vagum, on both sides, destroys an animal, say the horse, in a few hours, by paralyzing the muscles about the larynx, and thus causing asphyxia. When death is thus produced, we cannot properly ascertain the real effects of the interruption of the nervous influence on the stomach, lungs, and other organs. M. Dupuy, therefore, in his experiments, opens the trachea of the horse, by which the animal is made to live from fifty to sixty hours. The gentleman in question, has laid some experiments lately before the Medical Society of Paris, in which the effects of the pneumo-gastric section are shown. Thus, the nerves were divided in both sides of a horse's neck, and portions cut away. Tracheotomy was then immediately performed. The animal was carefully examined at certain periods, and also bled before and after the operation, in order to ascertain the effects of the section on the blood, as well as on various functions. Two hours after the section, there was no alteration in the functions of respiration, circulation, &c. The animal continued to eat as before. In four hours, the breathing was accelerated—the blood from the carotid was now darker in colour than before—the animal ate and drank, but deglutition seemed performed by a convulsive action, and liquids returned by the wound in the trachea. At the end of 16 hours, the breathing was slow and deep—much mucous flowed from the wound in the trachea, and the food returned by the same aperture—the action of the heart is much weakened—arterial blood is now nearly as dark as venous. The fourth examination, at the end of 28 hours, showed no material alteration. At 40 hours from the operation, the animal had great difficulty in swallowing—the blood from an artery was quite black—the œsophagus was felt to be crammed with food—and the horse was comatose. At 52 hours, the breathing was stertorous, and he soon died.

*Dissection.* There was nothing wrong in the brain. All the parts forming the aperture in the larynx were swelled and red, so that the passage was almost entirely closed. The mucous membrane of the larynx and trachea was red, and otherwise discoloured, but these were considered cadaveric phenomena. The lungs were inflamed and hepatised—the bronchia were filled with mucosities. The substance of the heart was much softened, and its cavities filled with black blood. The œsophagus was filled with alimentary matters, as were the pharynx and nasal cavities. The stomach was filled and distended with food almost dry, and adherent to the mucous membrane of the organ, which was of a red colour. There was no chyme in any of the intestines. The liver was enlarged, and there were several large black spots on the spleen.

From the above, and other experiments of a similar kind, it is evident that section of the pneumo-gastric nerves stops the sanguifictive (arterializing) process in the lungs—paralyzes the œsophagus --and puts an end to the secretion of gastric juice, and, consequently, digestion, in the stomach.

The experimenter, found, in this, and several other cases, that a peculiar effect is produced on the spleen by these operations—namely that its blood is changed, and capable of *producing* a disease in other animals, when introduced by a puncture, which affects *their* spleens and causes death. The same was found to be the case with the spleens of animals living in marshy countries, where intermittents prevail.—*Ibid.*

13. *On the Effects of Cupping Glasses on the Developement of Vaccine Pustules.* [*Journ. Gen. de Med. Mars*, 1828.]—The members of the Academie Royale de Medecine have been, for some time, endeavouring to prove the effects of exhausted glasses on virus and poison inserted into the skin, but they do not appear to have yet arrived at any satisfactory conclusion. Did Dr. Barry's theory of absorption lead to so useful a point in practice as to enable us to arrest the progress of poison inserted into the skin, it would be worthy of some attention, however contrary to the laws of physic it might appear. Dr. Barry, conceiving that the absorbent fluids are driven up towards the heart by the weight of the atmosphere on the surface of the body, was naturally led to suppose that, by depriving any part of that surface of atmospheric pressure, no absorption could go on in that part whilst so deprived. Facts, so far as they have yet been collected, tend to support this opinion, although they prove no more that the fluid in the absorbent vessels is propelled by the weight of the superincumbent atmosphere, than the flow of blood out of a punctured vein proves that the atmosphere *attracts* the fluid, against its own gravity, out of the punctured vessel. The committee appointed by the Academie to examine Dr. Barry's experiments, found that no absorption went on under a vacuum; that, in fact, if poison be inserted into the skin, and a cupping glass, with the air exhausted from it, be applied over the part, the absorption of that poison into the system is prevented. M. Itard, wishing to satisfy himself of this fact, repeated the experiment, not by inserting poison, as Dr. Barry did, but by inoculating the part with the vaccine virus. He vaccinated a child on the shoulders by several punctures, over some of which he applied glasses, whilst the rest were left uncovered. The punctures which were covered by the glasses formed no pustules, whereas all those which had not been so covered, gave rise to vaccine pustules, possessing the usual characters. M. Bousquet was not satisfied with the result of M. Itard's experiment, so that he determined to repeat it on a large scale, at the bureau of public vaccination of the Academie. M. Bousquet obtained a very different result from that which M. Itard had obtained. In the experiments of the former, the glasses appeared to have scarcely any sensible effect in preventing the puncture from forming a pustule. It appears to be the general opinion among the members of the Academie, that a vacuum produced over the puncture may prevent the action of poison on the system, which generally takes place very suddenly, and in a degree proportioned to the dose; but that it is different with regard to a *virus*, which has the property of reproduction, and whose action is slow.

This subject appears to us easily explained. The application of an exhausted glass to a part will suspend the function of absorption in that part while it remains on. It acts in two ways in doing this; first, by the pressure of its edge on the absorbing vessels running from the part, thereby obstructing the motion of their contents; second, by *expanding these vessels beyond their natural calibre*, thereby suspending their function for the time. The absorbents are naturally intended to sustain the weight of the atmosphere, and when this weight is removed, they necessarily expand, and assume a calibre which is not natural to them. The arteries and veins do the same. It cannot be expected that the absorbents, while in this state, can bring their sides together in order to press forward their contents. Let it be tried whether a portion of the intestinal canal will be able to contract so in a vacuum. As the vacuum of a cupping glass suspends the tonicity or contractility of the blood-vessels where it is applied, nothing can be more probable than that it suspends that of the absorbent vessels, whose coats are much more delicate than those of the arteries and veins. The fact appears to be, that the pressure of the atmosphere is natural to all the vessels, as it is to all other terrestrial objects, and that it is one of the causes which determine their physical form; it is also one of the causes which enables them to perform their functions, but it does not follow from this that they act like syphons, more than it does that the intestinal tube acts so.

Now it is not only possible, but also probable, that all the poison, or even virus, may be extracted, in some instances, if an exhausted glass is applied *immediately* after its introduction—that is, if the glass is applied before the poison or virus has entered the extremities of the absorbents. It may, by this means, be washed out by the blood. But should it once enter the extremities of the absorbents, nothing but a retrograde action of these vessels could discharge it again; for, as the edge of the glass extends all round to some distance beyond the wound, and as there is no pressure between the glass and the wound to force the poison back, it must necessarily remain at rest there, unless it can run back under to the law of gravity. The only chance of abstracting the poison when it has once entered the extremities of the absorbents, would be, by using a very small glass, which would barely extend beyond the edges of the wound. It is generally supposed, that, because an exhausted cupping glass extracts blood from a puncture, it must also extract the contents of the absorbents, or any thing else which may be in the wound. But this is by no means the case. The blood is constantly forced by the action of the heart towards that part, as well as towards others; it therefore flows out at the puncture. But as there is nothing to force the contents of the absorbents in a retrograde direction, that portion of the fluid situated between the edge of the glass and the wound will remain at rest, but the fluid situated between the glass and the heart may, and probably does, flow on in its natural course. It is true that the absorbents running into the wound from the distal side of the glass may pour out their contents, but it is not probable that any of the poison enters the divided extremities of these.

Now it would be advantageous to apply immediately an exhausted glass over the bite of a rabid animal, in order to suspend the absorption of the virus until instruments could be procured, and the consent of the patient and his friends obtained, to have the part excised. The absorption of any other virus or poison might be suspended in the same way for a period, to allow time to decide upon the best plan of treatment to be pursued. But should excision be considered necessary, all the parts which have been covered by glass should be removed before the patient can be considered safe.—*Lon. Med. and Surg. Journal, No.* 1.

